# Two Dimensional Electrostrictive Field Effect Transistor (2D-EFET): A sub-60mV/decade Steep Slope Device with High ON current

**DOI:** 10.1038/srep34811

**Published:** 2016-10-10

**Authors:** Saptarshi Das

**Affiliations:** 1 Department of Engineering Science and Mechanics & Material Research Institute, Pennsylvania State University, State College, 16803, USA

## Abstract

This article proposes a disruptive device concept which meets both low power and high performance criterion for post-CMOS computing and at the same time enables aggressive channel length scaling. This device, hereafter refer to as two-dimensional electrostrictive field effect transistor or 2D-EFET, allows sub-60 mV/decade subthreshold swing and considerably higher ON current compared to any state of the art FETs. Additionally, by the virtue of its ultra-thin body nature and electrostatic integrity, the 2D-EFET enjoys scaling beyond 10 nm technology node. The 2D-EFET works on the principle of voltage induced strain transduction. It uses an electrostrictive material as gate oxide which expands in response to an applied gate bias and thereby transduces an out-of-plane stress on the 2D channel material. This stress reduces the inter-layer distance between the consecutive layers of the semiconducting 2D material and dynamically reduces its bandgap to zero i.e. converts it into a semi-metal. Thus the device operates with a large bandgap in the OFF state and a small or zero bandgap in the ON state. As a consequence of this transduction mechanism, internal voltage amplification takes place which results in sub-60 mV/decade subthreshold swing (SS).

Ever since the inception of metal oxide semiconductor field effect transistor (MOSFET), Scaling has been the primary driving force behind its unprecedented success. The early era of scaling (∼1975–2005: Dennard Scaling) had two characteristic features: dimension scaling which allowed the number of transistor per chip to increase by 1000000x and consequently their speed to increase by 1000x, and voltage scaling which kept the power density practically constant throughout this scaling regime^1^,^2^. However, around 2005, the voltage scaling almost stopped as further reduction in the supply voltage (V_DD_) and hence the threshold voltage (V_TH_) was leading to exponential increase in the OFF state current (I_OFF_)^3^. This is a direct consequence of non-scalability of the subthreshold swing (SS) to below 60 mV/decade arising out of Boltzmann statistics that governs the operation of conventional MOSFETs. Dimension scaling, however, continued beyond 2005, but, under the new generalized scaling rules. This inevitably led to increase in the power density at the same rate as the integration density. The actual scenario is made worse by non-scaling factors which escalated static and leakage power densities at a much faster rate^3^. Power/heat dissipation, henceforth, became the main problem for high performance microprocessors. Today, in 2016, even dimension scaling seems extremely challenging beyond 10 nm gate length (L_G_) owing to fundamental material limitations. So it is not too far when all aspects of MOSFET scaling will completely stop, marking the end of the silicon complementary metal oxide semiconductor (CMOS) era. Therefore, in order to restore the golden era of transistor scaling, energy efficient and high performance innovative device ideas based on aggressively scalable novel materials need to be conceived on an urgent and immediate basis.

From the above discussion it is obvious that post-Si-CMOS devices have to resolve two key challenges: length scaling and voltage scaling. For length scaling, low dimensional systems like nanotubes^4^,^5^, nanowires^6^,^7^ and very recently nanosheets^8^,^9^,^10^,^11^,^12^,^13^,^14^ are being considered as alternative materials to Si due to their inherent electrostatic integrity that allows fundamentally superior scaling properties. Voltage scaling, however, necessitates steep slope devices which in turn require operation beyond Boltzmann statistics. Several steep switching device concepts like tunneling FETs^15^,^16^, piezoelectric strain modulated Si FinFETs^17^, negative capacitance ferroelectric FETs^18^ excitonic FETs^19^ and spin-based FETs^20^,^21^ have been proposed. Among these, tunneling FETs are the most matured candidates which have experimentally demonstrated SS less than 60 mV/decade^15^. However, the greatest challenge for tunneling FETs are their low ON state current densities limited by large tunneling barriers. Piezoelectric strain modulated Si FinFETs are also promising but suffers from the limitation of bulk nature of Si at the scaling limits. The readers should note that the objective for post-CMOS device design is mainly two-fold: SS slope should be as abrupt as possible (ideally zero) to meet the low power requirement whereas the ON current should be as high as possible to increase device speed.

In this context, 2D layered semiconductors are receiving significant attention as possible candidates for post-Si electronics owing to their ultra-thin body nature that allows aggressive channel length scaling and hence high performance. Moreover, recent experimental and theoretical studies show that the bandgap of multilayer transition metal dichalcogenides (TMDs: an extensively studied class of 2D materials) like MoS_2_, WSe_2_ etc. can be dynamically reduced to zero by applying out-of-plane stress^22^,^23^. In this article we, therefore, combine the superior scalability of 2D materials with stress induced dynamic bandgap engineering into a breakthrough device concept called Two Dimensional Electrostrictive Field Effect Transistor or 2D-EFET. As shown in Fig. 1, the device structure resembles a conventional 2D-FET with the exception that the gate insulator is substituted with an electrostrictive material. The operating principal, however, differs significantly since an electrostrictive material not only behaves like a high-k insulating gate oxide but also undergoes longitudinal expansion when an electric field is applied across it. This expansion transduces an out-of-plane stress on the 2D channel material and monotonically reduces its bandgap to zero. In the OFF state, the 2D-EFET operates with a large bandgap and prevents current conduction whereas in the ON state, it operates with a smaller or zero bandgap and allows current conduction. The fascinating aspect of 2D-EFET is that it offers steep SS below 60 mV/decade owing to an internal feedback mechanism giving rise to voltage amplification and at the same time provides significantly higher ON state current density compared to any existing charge based device concepts.

The operation of 2D-EFET is conceptually explained in Fig. 2 and through equation 1 through 8. Figure 2a shows the strain (S) *versus* electric field (ξ) characteristics (equation 1) of an electrostrictive material and Fig. 2b shows the bandgap (E_G_) *versus* out-of-plane stress (P) characteristics (equation 2) of a 2D material based on phenomenological models. The parameter sets (α, β, δ) and (E_G0_, κ, χ) are specific to the choice of electrostrictive and 2D material and their values used for our simulations were extracted from experimental literature (the linear approximation of equation 1 and 2 which are also shown in Fig. 2a,b will be used primarily for easier qualitative discussion). In 2D-EFET, equations 1 and 2 are coupled through equation 3, where, t_E_ is the thickness of the electrostrictive material, ϒ_2D_ and t_2D_ are, respectively, the Young’s modulus and thickness of the 2D material, and finally, η quantifies the ratio of strain transfer from the electrostrictive material to the channel material such that Δt_2D_ = η.Δt_E_ (0 < η < 1). Note that η is a very important parameter for the 2D-EFET since it determines the efficiency of strain transduction and hence the improvement in SS. It remains to be experimentally demonstrated how electrostrictive material can be integrated with 2D materials for maximum strain transfer. One possible strategy would be capping/encapsulating the entire device structure in a rigid fixture to minimize the expansion of the electrostrictive material away from the 2D channel. Other possible approach will be engineering better electrostrictive material which can provide higher strain than PMN-PT and thereby compensate for poor coupling efficiency. Recently, there has been significant progress towards the development of lead-free ceramics by various doping and alloying routes which includes titanates, alkaline niobates and bismuth perovskites and their solid solutions^24^. Specifically, giant electric field-induced strain (EFIS) was observed in Nb modified lead-free Bi_0.5_Na_0.5_TiO_3_ - Bi_0.5_K_0.5_TiO_3_ - LiTaO_3_ (BNT-BKT-LT) ternary system. With 3 mol% Nb substitution, the EFIS could be enhanced up to 641 pm/V^25^. Lead-free Zr-modified Bi_0.5_(Na_0.78_K_0.22_)_0.5_TiO_3_ ceramics (BNKTZ-100x, with x = 0–0.05) also demonstrated enhanced unipolar field-induced strain of 0.43%^26^.

Figure 2c shows an equivalent capacitive network model for our 2D-EFET where, V_GS_ is the applied external gate bias, Ψ_S_ is the electrostatic surface potential and C_E_, C_CH_ and C_IT_ are respectively the capacitances associated with the electrostrictive material, the 2D channel material and the interface traps. Figure 2d shows position of the energy bands inside the 2D channel material corresponding to the OFF and ON state of device operation. Note that the additional band movement Ψ_E_ appears due to the decrease in the bandgap of the channel material through electrostrictive transduction. Theoretical simulations performed on bilayer TMDs by Kumar *et al.*^23^ suggests that the conduction band (CB) minima between the K-Γ high symmetry point and the valence band (VB) maxima at the Γ point in the Brillouin zone of various TMDs move towards their corresponding Fermi levels when the inter-layer spacing between the successive van der Waals layers are monotonically reduced. It is also apparent from their calculations that the rate of movements of CB minima and VB maxima as a function of the inter-layer spacing are almost similar which justifies the assumption Ψ_E_ = 1/2   ΔE_G_. Note that the total channel potential (Ψ_T_ = Ψ_S_ + Ψ_E_) is always greater than Ψ_S_ and results in an internal voltage amplification. Figure 3 shows electrostatic (Ψ_S_), electrostrictive (Ψ_E_) and total channel potential (Ψ_T_) as a function of external gate bias (V_GS_) obtained by solving equation 4 through 6 self-consistently with equation 7. Note that C_CH_ enters into the expression for Ψ_S_ and Ψ_T_ through the quantity r. In equation 7, D(E) denotes the 2D density of states derived from parabolic energy dispersion relationship, m^*^ is the carrier effective mass, h is the Planck’s constant and f_S_(E) and f_D_(E) are the Fermi function for the source and drain contact electrodes, respectively. Finally equation 8 (k_B_ is the Boltzmann constant, q is the electronic charge and T is the temperature) represents the subthreshold swing (SS).


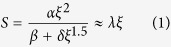



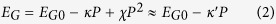



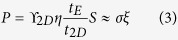



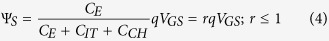



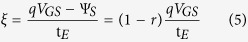



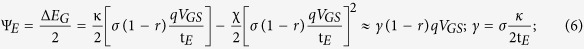



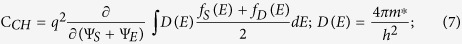



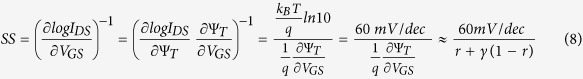


As evident from equation 8, in order to obtain sub-60 mV/decade SS, it is necessary to make ∂Ψ_T_/∂V_GS_ > 1 in the OFF state of the device operation. This is possible if and only if the quantity r < 1 which could be achieved through a finite value of C_CH_ or C_IT_ or both. Now, in a conventional 2D-FET (where γ = 0 and, therefore, Ψ_E_ = 0 and Ψ_T_ = Ψ_S_), C_CH_ is negligible in the OFF state since D(E) ≈ 0 and C_IT_ should ideally be negligible so that r = 1 (i.e. Ψ_S_ = V_GS_, in equation 4) and hence SS = 60 mV/decade. This is the best possible SS achievable within Boltzmann limit. In 2D-FETs r < 1 leads to SS > 60 mV/decade which is undesirable. But, this scenario is completely different for the proposed 2D-EFET, since r = 1 results in ξ = 0 and hence Ψ_E_ = 0. So no transduction leading to internal voltage amplification can occur. As shown in Fig. 3a, if C_IT_ = 0, the electrostatic band movement is almost one to one with the applied gate bias in the OFF state of the device (V_GS_ < V_FB_ − *v*_TB_, where, V_FB_ is the flat band voltage and *v*_TB_ denotes the thermal broadening; *v*_TB_ = 6k_B_T. We have used V_FB_ = 0.4 V for our simulations). This is true irrespective of the value of γ. However, as the device enters near threshold or ON state operation, C_CH_ becomes finite leading to r < 1 and Ψ_E_ > 0 as shown in Fig. 3a,b respectively. This initiates an internal feedback mechanism which requires a readjustment of Ψ_S_ since C_CH_ depends on both electrostatic and electrostrictive potential (equation 7). Hence, Ψ_S_ becomes non-monotonic in the ON state of device operation as shown in Fig. 3a. Note that higher values of γ are associated with more efficient strain transduction and hence stronger and more abrupt feedback mechanism. The total channel potential Ψ_T_, however, increases monotonically according to Fig. 3c. But, none of these scenarios are conducive for achieving sub-60 mV/decade SS since the internal voltage amplification (Ψ_T_ > Ψ_S_) takes place only in the ON state of the device operation. As shown in Fig. 3d–f, more favorable conditions can be achieved when C_IT_ is finite. This allows r < 1 and hence Ψ_E_ > 0 in the OFF state of the device operation which ultimately leads to internal voltage amplification (Ψ_T_ > Ψ_S_). However, the criterion for sub-60 mV/decade SS i.e. ∂Ψ_T_/∂V_GS_ > 1 is achieved only when γ > 1 and r < 1 as obvious from equation 9. Note that internal voltage amplification leading to sub-60 mV/decade SS can also be achieved in negative capacitance ferroelectric FETs (NCFETs). However, the working principle of NCFET is distinctly different compared to 2D-EFET^18^.

Figure 4a,b shows the transfer characteristics of 2D-EFET obtained by solving equations 4 through 7 self consistently with ballistic Landauer formalism^27^ (equation 9).


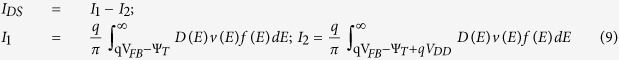


In equation 9, I_1_ and I_2_ are the current due to electron injection from the drain and the source contacts, respectively and v(E) is the carrier velocity. As expected sub-60 mV/decade SS is achieved for finite value of C_IT_ and γ > 1. Figure 4c shows the average SS (over 4 decades) as a function of γ for different values of C_IT_. It is counter intuitive from a conventional 2D-FET stand point to note that higher values of C_IT_ allow better subthreshold slopes in 2D-EFET for a given value of γ. But the reader should realize that although finite C_IT_ is detrimental for electrostatic band movement, it is beneficial for electrostrictive band movement. This is because larger C_IT_ ensures larger potential drop across the electrostrictive gate material which culminates into higher stress and greater bandgap reduction in the 2D channel material. In principle, if we make r = 0 corresponding to an infinitely large C_IT_, then Ψ_S_ = 0, which completely stops the electrostatic band movement in the channel. However, Ψ_E_ will still be finite due to the electrostrictive band movement. As long as ΔΨ_E_ > ΔV_GS_, internal voltage amplification will occur leading to SS < 60 mV/decade. Note that the improvement in the SS in 2D-EFET is essentially determined through the quantity γ which includes several material parameters corresponding to both the 2D material and the electrostrictive material. A larger overall value for γ will ensure a better SS as evident from equation 8. A thicker channel material will certainly make γ small in accordance with equation 6, whereas monolayer materials obviously lack van der Waals spacing. Bilayer materials are optimum for 2D-EFET since these are the thinnest with a finite van der Waals spacing. In the manuscript we have used the material parameters corresponding to MoS_2_ since it has been most extensively studied in experiments. Other TMDs also demonstrate similar effects, however there is lack of experimental literature. In fact as shown by Kumar *et al.*^23^, similar bandgap changes can be obtained at smaller strain values for MoSe_2_ and MoTe_2_ i.e. these materials offer larger κ values and hence larger γ.

Figure 4d shows the dynamic bandgap change as a function of the applied gate bias and Fig. 4e shows the output characteristics for the 2D-EFET with γ = 2.0 and C_IT_ = 3C_E_. The reader should remember that the length of the plateau in the output characteristics which is referred to as the saturation regime is equal to the bandgap of the channel material (at T = 0 K and shortened by few k_B_T due to thermal broadening at finite T) for a ballistic transistor. Since the bandgap changes in our 2D-EFET as a function of the applied gate bias, the length of the saturation region also changes accordingly in Fig. 4e. Figure 4f shows the ON current (I_ON_) and ON to OFF current ratio (I_ON_/I_OFF_) for the 2D-EFET as a function of the flat band voltage (V_FB_) for different supply voltages (V_DD_). As expected I_ON_ increases with increase in V_DD_ but decreases with increase in V_FB_, whereas, I_OFF_ remains practically constant with V_DD_ and decreases exponentially with increase in V_FB_ resulting in the trends observed in Fig. 4f. Clearly, the standard requirement for high performance FETs (i.e. I_ON_ = 1 mA/μm and I_ON_/I_OFF_ = 10^4^) can be achieved by 2D-EFET for V_DD_ = 0.2 V which is 3 times smaller than the predicted V_DD_ = 0.6 V for 2020 by ITRS. Moreover, ON current as high as 10 mA/μm can be delivered by 2D-EFET for a supply voltage of V_DD_ = 0.4 V. One criticism could arise from achievable operating speed in 2D-EFET since mechanical motions are slow and limited by the acoustic velocities. However, a simple back of the envelop calculation (conservative) with t_E_ = 100 nm and *v*_e_ = speed of sound in solid = 1000 m/s results in ~100 GHz operating speed. One obvious way to increase the speed would be the scaling of the thickness of the electrostrictive material (t_E_), however, this may lead to reduction in strain transfer and hence increase in SS. Optimization will be investigated in future studies. Finally, channel length (L_G_) scalability of 2D-EFET is determined through the band bending length λ (equation 10), which is derived by solving 2D Poisson’s equation similar to conventional planar FETs^6^. Therefore, from a pure electrostatic point of view, the use of ultra-thin 2D channels (t_2D_ = 1 nm, ε_2D_ = 8), with ultra-high-k dielectric (t_E_ = 100 nm, ε_E_ = 2000) allows scalability (L_G_ > 3λ = 1.8 nm) well beyond 10 nm technology node^6^.


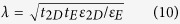


In the conclusion, a novel and disruptive device concept called 2D-EFET based on strain transduction and dynamic bandgap engineering in 2D material has been proposed and numerically evaluated. The 2D-EFET provides a breakthrough solution for post silicon, ultra-low power, high performance and aggressively scalable device technology.

## Additional Information

**How to cite this article**: Das, S. Two Dimensional Electrostrictive Field Effect Transistor (2D-EFET): A sub-60mV/decade Steep Slope Device with High ON current. *Sci. Rep.* **6**, 34811; doi: 10.1038/srep34811 (2016).

## Figures and Tables

**Figure 1:**
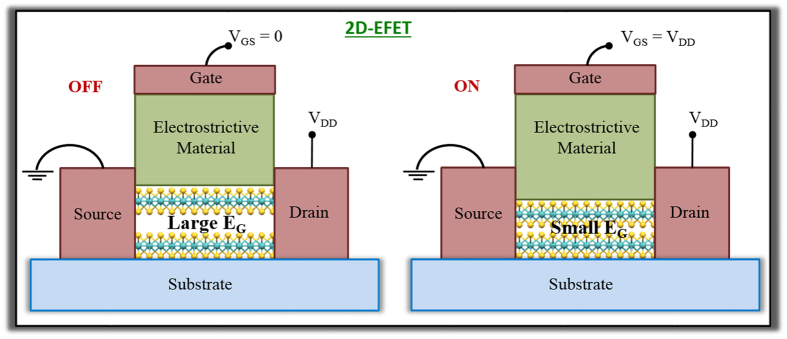
Proposed 2D-EFET. In the OFF state the 2D channel material offers a large bandgap and restricts current flow whereas in the ON state the 2D channel material offers a small or zero bandgap and allows current flow. The expansion in the electrostrictive material in response to the applied gate bias is compensated by reduction in inter-layer distance between two consecutive layers of the 2D material. This transduction results in an internal voltage amplification which allows the SS to become less than 60 mV/decade.

**Figure 2:**
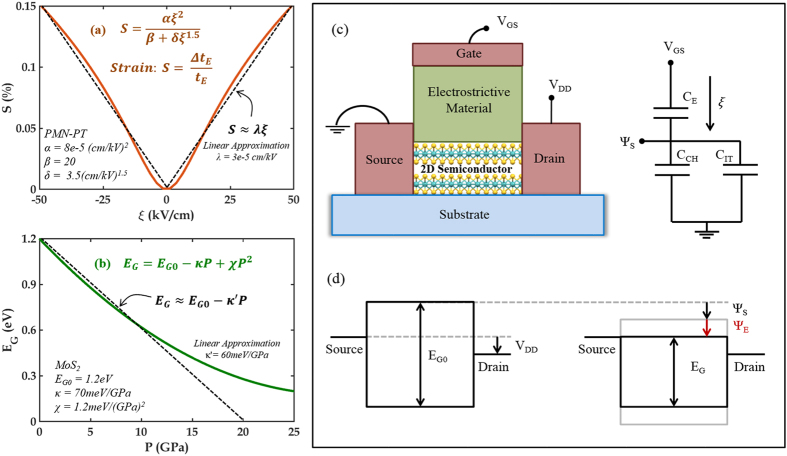
Operating principle for 2D-EFET. (**a**) Strain (S) *versus* electric field (ξ_e_) characteristics of an electrostrictive material (PMN-PT). (**b**) Bandgap (E_G_) *versus* out-of-plane stress (P) characteristics of a 2D material (MoS_2_). (**c**) Equivalent capacitive network for a 2D-EFET. (**d**) Schematic showing the band movement in 2D-EFET in response to applied gate bias (V_GS_). Ψ_S_ is the usual electrostatic component, whereas Ψ_E_ is the electrostrictive component. Ψ_E_ arises due to the reduction in the bandgap of the 2D material in response to the out-of-plane stress transduced by the electrostrictive material. This gives rise to internal voltage amplification which is ultimately responsible for steep subthreshold swing (SS) less than 60 mV/decade in 2D-EFETs.

**Figure 3:**
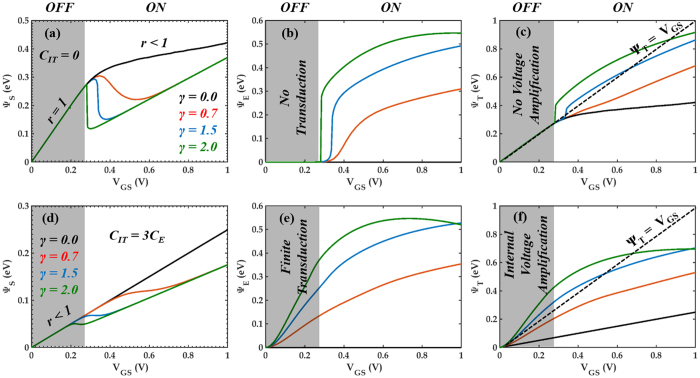
Channel Potential Map. (**a**) Electrostatic (Ψ_S_), (**b**) Electrostrictive (Ψ_E_) and (**c**) Total channel potential (Ψ_T_) as a function of applied gate bias (V_GS_) for γ = 0.0 (black), γ = 0.7 (red), γ = 1.5 (blue) and γ = 2.0 (green) with C_IT_ = 0. Clearly, the electrostatic band movement is one to one (r = 1) with V_GS_ in the subthreshold regime (OFF state) of the device operation and as such no electrostrictive transduction occurs. However, as the device enters near threshold or ON state operation feedback mechanism begins between electrostatic and electrostrictive potentials since r < 1 owing to finite channel capacitance (C_CH_). This leads to internal voltage amplification (Ψ_T_ > V_GS_) in the ON state as shown in Fig. 3c. The dotted black line represents Ψ_T_ = V_GS_. However, this amplification is not conducive for sub-60 mV/decade SS. Figure (**c–e**) shows respectively Ψ_S_, Ψ_E_ and Ψ_T_ as a function of V_GS_ for same values of γ but with finite C_IT_ = 3C_E_. In these instances, electrostrictive transduction take place even in the OFF state. However, internal voltage amplification only occurs with γ = 1.5 (blue) and γ = 2.0 (green). The material parameters used for our self-consistent simulations are as follows: α = 8e-5 (cm/kV)^2^, β = 20, δ = 3.5(cm/kV)^1.5^, E_G0_ = 1.6 eV, κ = 70 meV/GPa, χ = 1.2 meV/(GPa)^2^, ϒ_2D_ = 300GPa, t_2D_ = 1 nm, t_E_ = 100 nm, m^*^ = 0.45m_0_. The values of η (0 < η < 1) were adjusted to obtain the different γ values.

**Figure 4:**
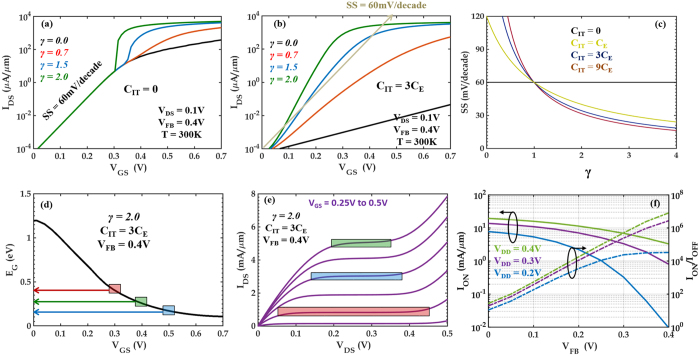
2D-EFET Device Characteristics. Room temperature current (I_DS_) *versus* gate voltage (V_GS_) characteristics of 2D-EFET for γ = 0.0 (black), γ = 0.7 (red), γ = 1.5 (blue) and γ = 2.0 (green) with (**a**) C_IT_ = 0 and (**b**) C_IT_ = 3C_E_. The η values used to obtain the corresponding γ values are 0.0, 0.2, 0.4, and 0.6, respectively. Source to drain bias of V_DS_ = 0.1 V and flat band voltage of V_FB_ = 0.4 V was used. (**c**) Subthreshold Swing (SS) as a function of γ for different values of C_IT_. Clearly sub-60 mV/decade SS is obtained if and only if C_IT_ > 0 and γ > 1. (**d**) Dynamic bandgap change in the channel of a 2D-EFET due to electrostrictive transduction for γ = 2, C_IT_ = 3C_E_ and V_FB_ = 0.4 V. (**e**) Room temperature output characteristics (I_DS_ *versus* V_DS_) for the same 2D-EFET for different values of V_GS_. The rectangular boxes in figure (**d,e**) correlate the length of the saturation region with the dynamic bandgap of the 2D-EFET. (**f**) ON current (I_ON_) and ON to OFF current ratio (I_ON_/I_OFF_) as a function of V_FB_ for different supply voltages (V_DS_ = V_GS_ = V_DD_). Ballistic Landauer formalism was used to compute the current *versus* voltage characteristics.
